# Development of a Fluorescent Rapid Test Sensing System for Influenza Virus

**DOI:** 10.3390/mi16060635

**Published:** 2025-05-28

**Authors:** Wei-Chien Weng, Yu-Lin Wu, Zia-Jia Lin, Wen-Fung Pan, Yu-Cheng Lin

**Affiliations:** Department of Engineering Science, National Cheng Kung University, Tainan 70101, Taiwan

**Keywords:** fluorescence immunoassay (FIA), image processing, influenza detection, optical detection, rapid test detection, sensing system

## Abstract

This paper presents a sensitive and stable fluorescence rapid test sensing system for the quantitative analysis of influenza rapid test results, integrating a detection reader to minimize errors from conventional visual interpretation. The hardware includes a control board, touchscreen, camera module, UV LED illumination, and a dark chamber, while the software handles camera and light source control, as well as image processing. Validation shows strong linearity, high precision, and reproducibility. For influenza A (H1N1), the system achieved a coefficient of determination (R^2^) of 0.9782 (25–200 ng/mL) and 0.9865 (1–10 ng/mL); for influenza B (Yamagata), the coefficient of determination (R^2^) was 0.9762 (2–10 ng/mL). The coefficient of variation ranged from 1–5% for influenza A and 4–9% for influenza B. Detection limits were 4 ng/mL for influenza A and 6 ng/mL for influenza B. These results confirm the system’s capability for accurate quantitative analysis while reducing reliance on subjective interpretation. Its compact, portable design supports on-site rapid testing and allows for potential expansion to detect other targets, such as COVID-19, RSV, and myocardial enzymes. The system’s scalability makes it a promising tool for clinical diagnostics, point-of-care testing (POCT), and infectious disease monitoring.

## 1. Introduction

Influenza is an acute viral respiratory infection that affects individuals of all age groups and poses a significant public health threat, especially during pandemics, seasonal epidemics, and sporadic outbreaks. Each year, approximately 10% of the global population is infected with influenza, resulting in an estimated 500,000 deaths worldwide [1]. Rapid and accurate diagnostic methods are essential for effective influenza management and outbreak control. Current diagnostic approaches include molecular techniques such as real-time polymerase chain reaction (PCR) [2], as well as immunoassay-based methods including rapid influenza diagnostic tests (RIDTs) [3], lateral flow immunoassays using colloidal gold [4], and lateral flow fluorescence immunochromatography (LFFC) [5]. Among these, LFFC combines the simplicity and rapidity of colloidal gold-based immunoassays with the high sensitivity of fluorescence-based detection [6]. This hybrid method not only enhances diagnostic sensitivity but also addresses some limitations associated with traditional fluorescence immunoassays, such as complex procedures, reagent instability, and storage challenges.

Influenza virus antigens (Ag) serve as the principal targets in rapid diagnostic testing. Also known as immunogens, antigens are recognized by the immune system upon viral entry, triggering an immune response characterized by the production of virus-specific antibodies. These antigens may originate not only from viruses, but also from bacteria or apoptotic cells within a host organism [7,8]. Antibodies (Ab), or immunoglobulins—most commonly IgG—are highly specific immune proteins that bind to antigens during the immune response. The antigen–antibody interaction forms the fundamental basis for immunological diagnostic techniques. A variety of analytical methods have been developed for the detection of viral antigens, with standard immunoassays including radioimmunoassay (RIA) [9], fluorescence immunoassay (FIA) [10,11,12], enzyme-linked immunosorbent assay (ELISA) [13,14,15], and chemiluminescence immunoassay (CIA) [16,17]. These immunoassay platforms are valued for their high sensitivity, which enables the detection of antigens present at low concentrations. They also ensure specificity in antigen–antibody recognition, reduce non-specific background signals, and provide rapid detection—often yielding results within minutes [18,19]. According to Onnerfjord et al., fluorescence polarization immunoassay (PFIA) enabled rapid analysis within 5 min, achieving detection limits as low as 0.08–0.4 ng/mL for atrazine, while effectively characterizing antibody specificity and cross-reactivity toward related triazine compounds [20].

Before the emergence of image-based interpretation technologies, medical diagnostics primarily relied on qualitative visual assessment or semi-quantitative evaluation using colorimetric charts [21,22,23]. These conventional methods offered limited clinical reliability, particularly in influenza detection, where accurate identification of low-concentration antigens is crucial. Misinterpretation in such cases can lead to false-positive or false-negative results, undermining diagnostic validity [24,25].

To meet the growing demand for precision and sensitivity in clinical diagnostics, the field has evolved toward quantitative analysis. This shift has driven advancements in both detection reagents and imaging-based colorimetric systems. Since the early 2000s, significant progress has been made in the development of image-assisted biomedical detection technologies [26,27], many of which have been successfully integrated into rapid diagnostic test platforms [28,29]. Das et al. developed a smartphone-based image sensing system that analyzes RGB values from colorimetric TB test spots using a feedforward neural network, achieving 87% sensitivity, 82% specificity, and 82% accuracy, thereby reducing human interpretation errors [30].

Fluorescence immunoassays (FIA), while offering superior sensitivity, require controlled optical conditions and differ fundamentally in detection mechanisms from traditional colorimetric methods [31,32,33,34]. Moreover, batch-to-batch variability in the manufacturing of fluorescent lateral flow test strips can lead to inconsistencies in signal development, particularly affecting the intensity of the test (T-line) and control (C-line) lines. As a result, reliable interpretation of FIA-based results necessitates concurrent evaluation of both lines, typically by calculating the fluorescence intensity ratio of the T-line to the C-line (T/C ratio) [35,36,37].

This paper presents the development and implementation of a fluorescence rapid test sensing system, designed to address the challenges of signal stability and interpretation encountered during fluorescence strip detection under general ambient lighting conditions. The system is built upon a design philosophy of operational simplicity and analytical accuracy, utilizing fluorescent lateral flow immunoassay test strips for the detection of influenza virus antigens [6,19]. A sealed optical chamber with light-blocking capability and a voltage-controlled UV LED illumination module is integrated to enhance fluorescence excitation efficiency and signal stability, enabling reliable operation in non-ideal, non-laboratory environments. To ensure signal consistency and reproducibility across different users and testing conditions, the system incorporates a real-time image acquisition module and an automatic T/C fluorescence intensity ratio computation algorithm.

Previous studies have suggested that fluorescence lateral flow analysis systems, although capable of achieving high sensitivity, often require relatively complex signal acquisition setups and external supporting hardware, which may increase system complexity and operational costs in certain applications [19]. To potentially address these concerns, the system presented in this work incorporates an embedded display and onboard data storage, allowing for a self-contained analytical process without the need for external computing equipment. This configuration supports on-site result interpretation and data recording. The system can be powered by rechargeable lithium batteries or portable power sources, which may facilitate its use in settings with limited infrastructure. Additionally, a modular hardware architecture—with adjustable illumination parameters, replaceable optical filters, and optional multi-channel configurations—could offer flexibility for future multiplexed detection tasks. Overall, the proposed approach may help reduce dependency on multiple instruments while improving adaptability, portability, and practical applicability in various point-of-care or field scenarios.

## 2. Materials and Methods

### 2.1. Fluorescence Strip

**Figure 1:**
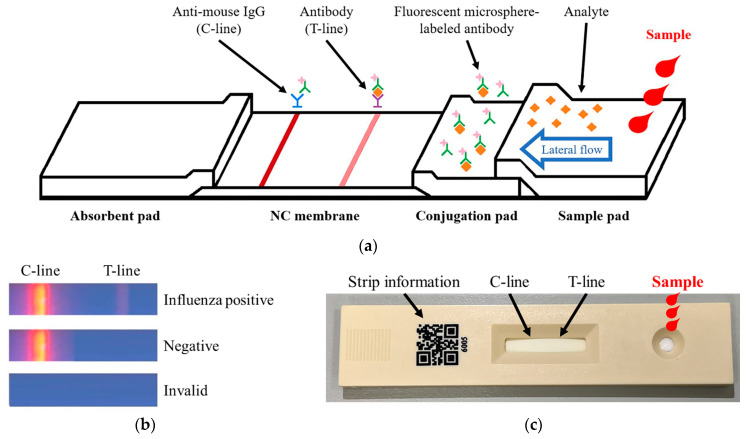
Fluorescence strip introduction. (**a**) Schematic diagram of the fluorescence strip structure. (**b**) Fluorescence signal development in the strip. (**c**) Physical representation of the fluorescence strip.

The schematic of the fluorescence strip used in this letter is shown in Figure 1a. The strip consists of four key components: sample pad, conjugation pad, nitrocellulose (NC) membrane, and absorbent pad, where capillary action enables passive sample flow and facilitates analysis. The sample pad serves as the initial contact point for the specimen, primarily functioning to filter the sample and remove interfering substances that may affect the immunoreaction. Additionally, by adjusting the material density, the sample pad regulates the flow rate, ensuring smooth migration of the specimen toward the reaction zone. As the sample reaches the conjugation pad, influenza virus antigens, if present, bind to pre-deposited fluorescent-labeled monoclonal antibodies, forming antigen–antibody complexes that migrate to the NC membrane. The NC membrane contains two detection zones: the T- and C-lines. The T-line is coated with monoclonal antibodies specific to influenza virus antigens. If these antigens are present, the complex binds and generates a fluorescence signal at the T-line (Figure 1b). The C-line, coated with anti-mouse IgG polyclonal antibodies, verifies assay validity by capturing fluorescent-labeled antibodies, generating a fluorescence signal regardless of the presence of the target antigen. The absorbent pad controls fluid flow and prevents backflow into the NC membrane, ensuring assay stability and accuracy. The physical structure of the strip is illustrated in Figure 1c. The right-side circular port serves as the sample loading zone, the central region contains the T- and C-line reaction area, and the left-side QR code stores strip information for scanning and digital processing. The strips used in this study include separate tests for influenza A and influenza B, following a 1C1T (one C-line, one T-line) format. Since the two test strips share an identical appearance, they can only be distinguished by scanning the QR code.

### 2.2. Hardware Architecture of the Detection Reader

**Figure 2:**
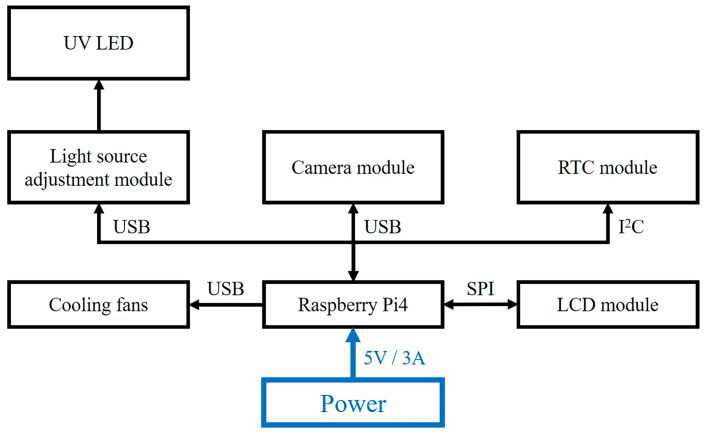
Hardware architecture of the detection reader.

The detection reader integrates a Raspberry Pi4 control board, an LCD touchscreen, a real-time clock (RTC) module, a USB visible light camera module (Model HB9080H13-S1-5A0, Fangtec corporation, New Taipei City, Taiwan), a light source adjustment module, 365 nm UV LEDs, and cooling fans, powered by a 5 V/3 A supply (Figure 2). The Raspberry Pi4 centrally manages signal transmission, image processing, and human–machine interface (HMI) control. The LCD touchscreen facilitates user interaction and real-time results display, while the RTC module ensures timestamp synchronization. The camera module captures fluorescence signals and scans QR codes for strip identification. The light source adjustment module receives signals from the control board and regulates the output LED intensity through current and voltage stabilization. The module is designed with multiple control modes, allowing future integration of different light sources for various rapid test applications. The cooling fans dissipate heat generated by the control board and other modules, ensuring stable operation of the detection reader and preventing potential impacts on light source intensity and detection accuracy.

### 2.3. Image Processing

**Figure 3:**
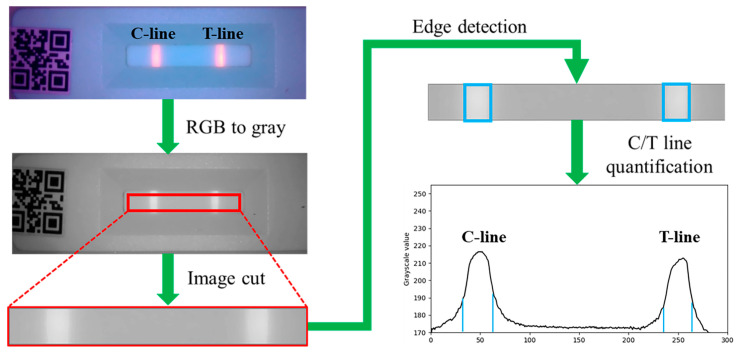
Image processing workflow for fluorescence strip.

The image processing workflow for fluorescence strip analysis begins with capturing an image using a camera module and storing it in RGB format (0–255 intensity range per channel). The RGB image is then converted into a grayscale matrix, followed by segmentation to isolate the C-line and T-line regions for analysis, as illustrated in Figure 3.

This paper employs the Python version 3.7 programming language in conjunction with the OpenCV library to develop an image processing algorithm. Experiments were conducted within a sealed dark chamber to eliminate variations in ambient lighting, ensuring consistent illumination conditions and precise reagent placement within designated reaction zones. To minimize noise interference, the camera module inside the dark chamber was operated using fixed parameters such as brightness, contrast, ISO sensitivity, exposure time, and resolution.

Each reagent used in this paper has a QR code printed at a fixed location, which serves both as an identifier for reagent type and as a spatial reference for image localization. After image acquisition, the position of the QR code is first identified, and this location is used to extract a predefined region of interest (ROI) for subsequent image processing and reagent recognition.

The identification process for the C-line and T-line is illustrated in Figure 3. Initially, the ROI image is converted to grayscale to eliminate color interference and improve computational efficiency. The average pixel intensity of the grayscale image is then calculated to establish a reference for background brightness. Using edge detection techniques, regions with grayscale values exceeding the background level are identified as potential C-line and T-line candidates. Finally, these candidate regions are classified as either C-line or T-line based on their spatial distribution relative to the QR code.

### 2.4. Quantitative Data Calculation (T/C Ratio)

During the image analysis process, it is essential to establish consistent standards for each test. Therefore, reagent images captured at each instance must be quantified and normalized. The first step in image processing is determining the background grayscale value. Subsequently, the grayscale intensities of the C-line and T-line are measured, with background subtraction applied to obtain their quantitative values. Due to batch-to-batch variations in fluorescence strip manufacturing, C-line intensity may fluctuate. To ensure standardization and normalization, each measurement is adjusted using the T/C fluorescence intensity ratio (T-line intensity divided by C-line intensity), enabling reliable data comparison across different fluorescence strips. For validation, fluorescence strips for influenza A (H1N1) and influenza B (Yamagata) were tested to assess the system’s linearity, sensitivity, and reliability.

## 3. Results and Discussion

### 3.1. Fluorescence Rapid Test Detection Reader

**Figure 4:**
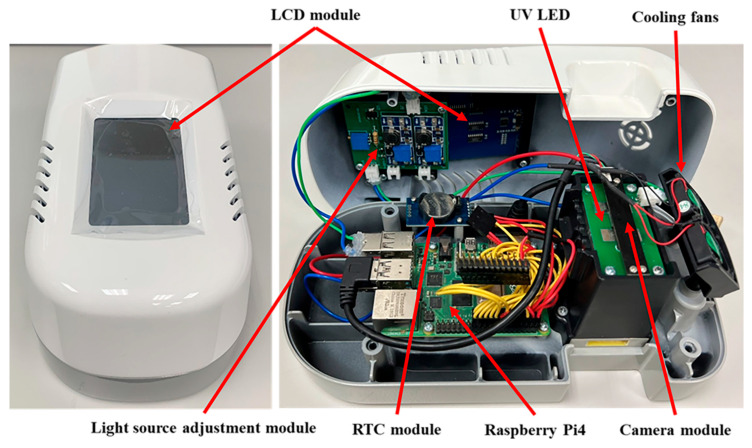
Physical appearance of the fluorescence rapid test detection reader.

After finalizing the hardware architecture of the fluorescence rapid test detection reader, its enclosure was fabricated based on the dimensions of each circuit module. A computer numerical control (CNC) machine was used for precise machining, followed by assembly and structural adjustments. The fully assembled detection reader is shown in Figure 4. The top cover integrates the LCD touchscreen and light source adjustment module. In contrast, the bottom cover houses the Raspberry Pi4 control board, RTC module, optical detection dark chamber, and cooling fan mounts.

### 3.2. UV LED Light Intensity Testing

**Figure 5:**
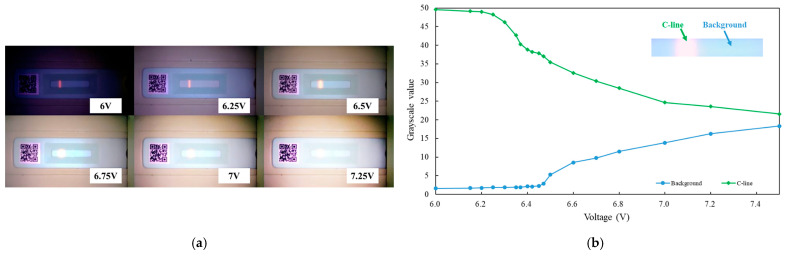
Experiment on UV LED control using the light source adjustment module. (**a**) Captured images of the strip at different voltages. (**b**) Grayscale data corresponding to voltage variations.

After integrating the hardware and circuit modules, tests were conducted on the voltage control of the light source adjustment module and the UV LED illumination. A fluorescence strip with only the C-line visible was used for evaluation. By adjusting the supply voltage of the light source adjustment module, the intensity of the UV LED was varied, and images of the fluorescence test strip under different voltages were captured using the camera module. The corresponding voltage values of the UV LED and the captured images of the test strip are shown in Figure 5a. When the voltage was set to 6 V, the QR code on the rapid test strip appeared excessively dark and became completely unrecognizable. Moreover, as the voltage continued to increase beyond 6.5 V, overexposure of the C-line on the test strip was observed in the captured images. Additionally, the data in Figure 5b show that the grayscale value of the C-line gradually decreased beyond 6.25 V, while the background grayscale value significantly increased after 6.5 V, with both values converging as the voltage increased. This result indicates that the excessive intensity of the UV LED, caused by the increased voltage, led to image overexposure, making it impossible to distinguish the C-line. Based on these findings, this study determined that the optimal voltage range for the UV LED is between 6.25 V and 6.5 V.

### 3.3. Influenza A Concentration Analysis

**Figure 6:**
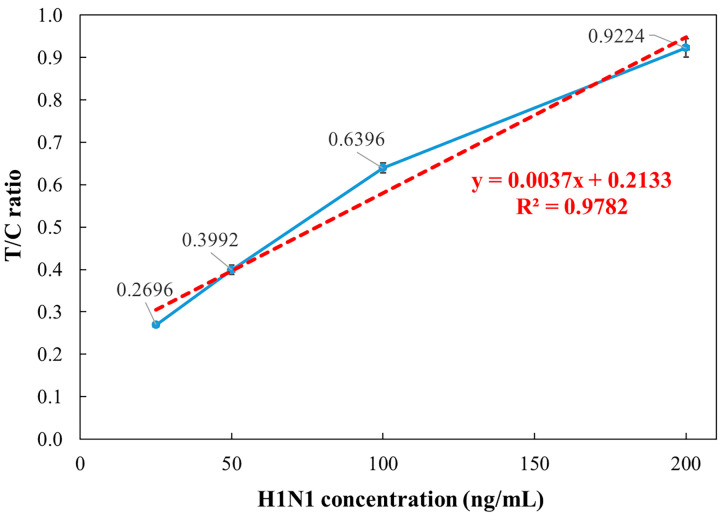
Relationship between influenza A (H1N1) concentration and T/C ratio over the 25–200 ng/mL range.

The detection reader was validated using standard samples of influenza A (H1N1) and influenza B (Yamagata). According to the World Health Organization (WHO) guidelines, Flu-IVDs belonging to the HA group were evaluated for their analytical sensitivity using the international standard influenza antigens. The analytical sensitivity of Flu-IVDs for A/H1N1 has been reported to be 500–1000 ng/mL [38]. Therefore, a concentration of 500 ng/mL was selected and subsequently diluted to 200, 100, 50, and 25 ng/mL for preliminary testing. For influenza A (H1N1), samples were prepared at concentrations of 200, 100, 50, and 25 ng/mL, with 130 μL applied to the fluorescence strip. After a 15 min incubation, fluorescence signals were measured. Each concentration was tested five times, recording T/C ratio to calculate the mean, standard deviation, and coefficient of variation (CV). As shown in Figure 6, the detection reader demonstrated strong linearity, with the coefficient of determination (R^2^) of 0.9782 in the 25–200 ng/mL range. The coefficient of variation CVs ranged from 1% to 3%, confirming high reliability.

### 3.4. Influenza A Low-Concentration Analysis

**Figure 7:**
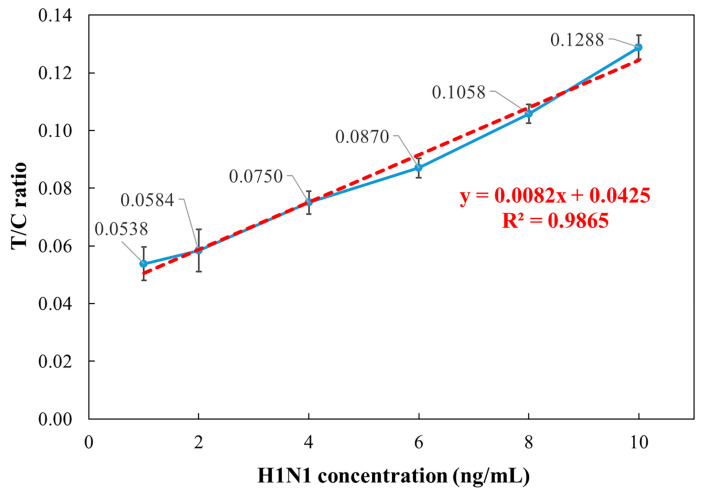
Relationship between influenza A (H1N1) concentration and T/C ratio over the 1–10 ng/mL range.

To evaluate the sensitivity of the detection reader at low concentrations, the influenza A (H1N1) standard samples were further diluted to 10, 8, 6, 4, 2, and 1 ng/mL, and the same measurement procedure was repeated. As shown in Figure 7, the results demonstrated a strong coefficient of determination (R^2^ = 0.9865) in the 1–10 ng/mL range. However, at 2 ng/mL and 1 ng/mL, the measured values were nearly overlapping, with CVs exceeding 10%, whereas the 4–10 ng/mL range exhibited CVs ranging from 3% to 5%. These findings suggest that the limit of detection (LOD) for influenza A (H1N1) using the detection reader is approximately 4 ng/mL, demonstrating high sensitivity in low-concentration analysis.

### 3.5. Influenza B Low-Concentration Analysis

To validate the performance of the detection reader, additional tests were conducted using influenza B (Yamagata) as the target. According to the World Health Organization (WHO) guidelines, Flu-IVDs belonging to the HA group were evaluated for their analytical sensitivity using the international standard influenza antigens. The analytical sensitivity of Flu-IVDs for Flu B has been reported to be 1000 ng/mL [38]. Therefore, a concentration of 1000 ng/mL was selected and subsequently diluted to 10, 8, 6, 4, 3, and 2 ng/mL for testing. For each concentration, 130 μL of the sample was applied to the fluorescence strip, incubated for 15 min, and measured using the detection reader.

**Figure 8:**
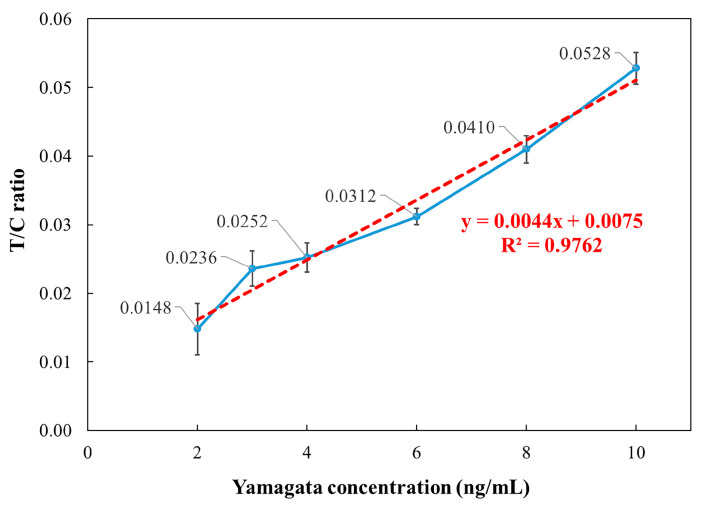
Relationship between influenza B (Yamagata) concentration and T/C ratio over the 2–10 ng/mL range.

Similar to the influenza A (H1N1) analysis, each concentration was tested five times, and the T/C ratio values were recorded to calculate the mean, standard deviation, and CV. The relationship between concentration and detection reader output is presented in Figure 8. The results indicate that the system demonstrated strong linearity, with the coefficient of determination R^2^ = 0.9762 in the 2–10 ng/mL range for influenza B (Yamagata). However, the CV ranged from 4% to 9% in the 4–10 ng/mL range, while at 3 ng/mL, the CV was 10.92%, and at 2 ng/mL, it increased to 25.43%, both exceeding 10%. Additionally, the measured values for 4 ng/mL and 3 ng/mL were nearly overlapping, indicating that the LOD for influenza B (Yamagata) using the detection reader is approximately 6 ng/mL. These findings confirm that the developed detection reader exhibits strong linearity and stability in concentration analysis of influenza B (Yamagata) standard samples.

### 3.6. Comparison with Commercial Detection Systems

**Figure 9:**
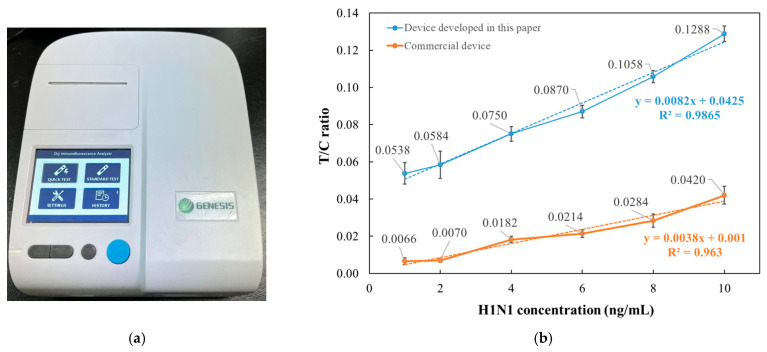
Comparison with commercial device. (**a**) Physical appearance of commercial device. (**b**) Comparison of data between the equipment developed in this paper and commercial device.

During the research, the commercial detection system used as a reference for comparison was the Dry Immunofluorescence Analyzer produced by GENESIS (Hangzhou, China), as shown in Figure 9a.

Compared to the commercial device, the system developed in this paper is approximately 40% smaller in size and more than 50% lighter in weight. Functionally, the commercial system relies on mechanical buttons for interface settings and requires the insertion of a dedicated IC chip to load reagent information. In contrast, our device features a touch screen and can automatically recognize QR codes embedded with reagent data, enabling fully automated operation. Additionally, the system in our device completes image analysis in just 10 s.

Regarding test performance, this section uses the Influenza A low-concentration test as a comparison example. As shown in Figure 9b, the data from the commercial device exhibits relatively low linearity and limited discriminative capability.

## 4. Conclusions

This paper presents the design and development of a fluorescence rapid test detection reader for detecting influenza A (H1N1) and influenza B (Yamagata). A comprehensive dataset was established by integrating optical detection and image processing, and the system’s linearity, reliability, and stability were validated. For influenza A (H1N1), the system achieved coefficient of determination (R^2^) values of 0.9782 in the 25–200 ng/mL range and 0.9865 in the 1–10 ng/mL range, with CVs ranging from 1% to 5%. For influenza B (Yamagata), the system demonstrated coefficient of determination (R^2^) values of 0.9762 in the 2–10 ng/mL range, with CVs ranging from 4% to 9%. Additionally, the LOD was determined as 4 ng/mL for influenza A and 6 ng/mL for influenza B.

Compared to commercial detection systems, the developed detection reader offers advantages in miniaturization, rapid processing, portability, and ease of operation. These features highlight its potential for broader applications in fluorescence strip detection. Future work will focus on expanding validation across different rapid test strips to assess the system’s accuracy and performance further.

## Data Availability

Data are contained within the article.
